# Covalent immobilization of microbial naringinase using novel thermally stable biopolymer for hydrolysis of naringin

**DOI:** 10.1007/s13205-015-0338-x

**Published:** 2016-01-06

**Authors:** Ghada E. A. Awad, Abeer A. Abd El Aty, Abeer N. Shehata, Mohamed E. Hassan, Magdy M. Elnashar

**Affiliations:** 1 Chemistry of Natural and Microbial Products Department, National Research Centre, Dokki, Giza, Egypt; 2 Biochemistry Department, National Research Centre, Dokki, Giza, Egypt; 3 Biomedical Sciences Department, Curtin University, Perth, Australia; 4 Polymers Department, National Research Centre, Dokki, Giza, Egypt; 5 Encapsulation and Nanobiotechnology Group, Center of Excellence, National Research Centre, Dokki, Giza, Egypt

**Keywords:** Naringinase, *Aspergillus niger*, Immobilization, ART-FTIR (total reflectance Fourier transform infrared), TGA (thermal gravimetric analysis) and biopolymer (grafted alginate gel beads)

## Abstract

Naringinase induced from the fermented broth of marine-derived fungus *Aspergillus niger* was immobilized into grafted gel beads, to obtain biocatalytically active beads. The support for enzyme immobilization was characterized by ART-FTIR and TGA techniques. TGA revealed a significant improvement in the grafted gel’s thermal stability from 200 to 300 °C. Optimization of the enzyme loading capacity increased gradually by 28-fold from 32 U/g gel to 899 U/g gel beads, retaining 99 % of the enzyme immobilization efficiency and 88 % of the immobilization yield. The immobilization process highly improved the enzyme’s thermal stability from 50 to 70 °C, which is favored in food industries, and reusability test retained 100 % of the immobilized enzyme activity after 20 cycles. These results are very useful on the marketing and industrial levels.

## Introduction

Microorganisms are used in various fields especially in industries due to their ability to produce various enzymes, among these enzymes is naringinase one of the great important enzymes (Deene and Lingappa 2013).

Naringinase (EC3.2.1.40) is a hydrolytic enzyme containing both α-l-rhamnosidase and β-glucosidase activities. Firstly, α-l-rhamnosidase hydrolyzes naringin into rhamnose and prunin (4,5,7-trihydroxy flavanone-7-glucoside), the prunin is then simultaneously converted into glucose and naringenin (4,5,7-trihydroxy flavanone) by the β-glucosidase activity (Yusof et al. 1990).

Naringinase is used in debittering of citrus juices, and naringinase also finds applications in the production of glycopeptide antibiotic, deglycosylation of flavonoids, and gellan depolymerisation (Kamiya et al. 1985; Puri et al. 2005). The hydrolysis products (rhamnose, prunin, and naringenin) showed biological activities and can be used as starting materials for the synthesis of substances applied in pharmaceutics, cosmetics, and food technology (Ellenrieder et al. 1998).

Naringin (4,5,7-trihydroxy flavanone-7-rhamnoglucoside) is a bitter flavonoid present in several citrus fruits, especially grapefruits (Puri and Banerjee 2000). Naringin is the source of undesirable bitterness especially in citrus fruit juice industry; therefore, it must be removed or reduced its levels from the processed products (Yusof et al. 1990; Hasegawa and Maier 1993). This bitter taste was decreased by the reduction of naringin content which was carried out using chemical methods, but this method had several drawbacks resulting in the inferior quality of fruit juice (Puri et al. 2005). Nowadays, microbial naringinase has completely replaced the chemical methods due to cost-effective production and economically viable process (Puri and Banerjee 2000).

Many carriers for enzyme immobilization are found in the literature; however, efficient commercial carriers suitable for immobilization of enzymes used in industries are few and relatively expensive (Bickerstaff 1995). Most carriers are based on synthetic or natural polymers. The immobilization technique would enable the reusability of enzymes for tens of times, easier product separation, reducing the enzyme and the enzymatic product’s cost significantly. To prepare a new carrier for enzyme immobilization, it is naturally an advantage if substances that are already permitted for use in the pharmaceutical or food industries can be utilized. Hydrogels such as alginates, carrageenan, and chitosan are polysaccharide families belonging to this category that are commercially available, have diverse features, and are available at a reasonable cost (Hugerth et al. 1997). One of the main disadvantages of these biopolymers is that they are usually used for immobilization of enzymes using non-covalent bonds due to the lack of functionalities, for example, immobilized glucoamylase using Ca-alginate gel coated with partially quaternized polyethyleneimine (Tanaka et al. 1984). Unfortunately, the entrapment of enzymes in hydrogels is often characterized by some diffusion of the biocatalyst from the support, particularly for enzymes with molecular weight less than 300 kDa (Tanriseven and Dogan 2002). Elnashar and Hassan (2014), Elnashar et al. (2013), and Awad et al. (2013) have completely studied the covalently bond immobilizing enzymes by using hydrogels.

In this work, naringinase produced by marine-derived fungus *Aspergillus niger* was immobilized onto grafted alginate gel beads which consider as thermally stable biopolymer to improve the enzyme stability to be convenient for different industrial applications and evaluation of the effects of immobilization on naringin hydrolysis.

## Materials and methods

### Chemicals

Sodium alginate (Alg) was obtained from Fluka. Polyethyleneimine (PEI), glutaraldehyde (GA), and naringin were obtained from Sigma-Aldrich Company. Other chemicals were of analar or equivalent quality.

### Microorganism and its maintenance

The marine-derived fungus *A*. *niger*, isolated from decayed wood samples of old ships, submerged in sea water of Ismailia, Egypt (Shehata and Abd El Aty 2014). Fungal isolate was routinely grown on malt extract agar medium at 27 °C and preserved at −80 °C in 50 % (v/v) glycerol (Höller et al. 1999).

### Cultivation conditions and crude enzyme extraction

The optimal combinations of the major constituents of media for maximal naringinase production were evaluated as follows: 15 g orange rind waste, 30 ml moisture content, 1 % grape fruit powder, 1 % NaNO_3_, 0.5 % KH_2_PO_4_, 5 mM MgSO_4_, 5 mM FeSO_4_, and pH 7.5. Flasks were autoclaved for 20 min at 121 °C and cooled to room temperature before inoculation (Shehata and Abd El Aty 2014). Sterilized solid-state fermentation was inoculated with 1.0 ml inoculum containing (5 × 10^6^ spores/ml) of 5-day-old culture. The contents of the inoculated flasks were well mixed and incubated at 28 °C for 10 days. At the end of incubation period, 50 ml of sodium acetate buffer 0.1 M, pH 4.0, was added to the cultures and placed on a rotary shaker for 30 min. The suspension was filtered through a nylon cloth, followed by centrifugation at 5000 rpm for 15 min, 4 °C. The filtrate obtained was used for determination of naringinase activity (Abd El Aty et al. 2014; Faten and Abeer 2013)

### Enzymatic assay of naringinase activity

Naringinase was assayed for its activity by measuring the rate of glucose formation from the two-step hydrolysis of naringin to prunin and rhamnose by α-l-rhamnosidase and to naringenin and glucose using by β-glucosidase. The reaction mixture consists of 0.8 ml of 0.2 % naringin solution in 0.1 M sodium acetate buffer, pH 4.0; 0.2 ml enzyme was incubated at 50 °C for 60 min. One ml of the reaction mixture was tested for the presence of glucose by dinitrosalicylic acid (DNS) method (Miller 1959). One unit of naringinase activity was defined as the amount of enzyme that liberates 1 µmole of reducing sugars, expressed as glucose, under the given assay conditions.

### Preparation and grafting of alginate beads (Alg/(Ca^2+^ + PEI)/GA)

Grafted gel beads were prepared according to Elnashar (2010) after slight modifications. Sodium alginate (Alg) was dissolved in distilled water to give a final concentration of 1.5 % (w/v). The alginate solution was dropped through a nozzle of 300 μm using the Innotech Encapsulator in a hardening solution containing 2.5 % (w/v) CaCl_2_ (Ca^2+)^ and 1 % PEI solution and was soaked for 3 h. After washing, the beads were soaked in GA solution of 2.5 % (v/v) for 3 h.

### Elucidation of the modified gels using ATR-FTIR

The attenuated total reflectance Fourier transform infrared has been used to identify the new functionalities on the grafted alginate gels. IR transmission spectra were obtained using a FTIR spectrophotometer (FTIR-8300, Shimadzu, Japan). The test is aiming to prove the presence of the new functional group, carbonyl group, formed at all the different formulas. A total of 2 % (w/w) of the sample was mixed with dry potassium bromide (KBr). The mixture was ground into a fine powder using an agate mortar before it was compressed into a KBr disk under a hydraulic press at 10,000 psi. Each KBr disk was scanned 16 times at 4 mm/s at a resolution of 2/cm over a wave number range of 400–4000/cm, using Happ-Genzel apodization. The characteristic peaks were recorded.

### Thermal gravimetric analysis (TGA)

Thermal gravimetric analysis was performed to prove the formation of a strong polyelectrolyte complex between the Alg and PEI followed by GA. The thermal behavior of the different gel formulations (Alg), (Alg + PEI), (Alg + PEI + GA), and (Alg + PEI + GA + Enzyme) was characterized by the TGA (SDT 600, TA Instruments, USA). Approximately 3–6 mg of the dried gels was weighed into an alumina pan. The samples were heated from 50 to 1000 °C at a heating rate of 10 °C/min.

### Immobilization of naringinase onto grafted gel beads

One gram of gel beads was incubated with 2 ml of naringinase solution in appropriate concentration for 24 h at 4 °C. At the end of incubation period gel, beads were washed twice with 0.1 M sodium acetate buffer, pH 4.0, and used for naringinase assay.

Assay for immobilized naringinase was carried out by adding one gram of the immobilized gel beads to 2 ml of 0.2 % naringin solution in 0.1 M sodium acetate buffer, pH 4.0, and the liberated glucose was estimated as cited before.

### Optimization of the enzyme loading capacity and loading time using grafted alginate beads

In this experiment, 1 g of gel beads was incubated in 2 mL of enzyme solution in different dilutions ranged from 10 to 100 %, enzyme dilutions were prepared in 0.1 M sodium acetate buffer pH 4. Enzyme solution and gel beads were incubated at 4 °C for 24 h. The enzyme loading capacity (E.L.C.) or the amount of enzyme units immobilized onto gel beads was calculated as follows:

1 $${\text{E}}.{\text{L}}.{\text{C}}. = (Mo - Mf)/W,$$

where *Mo* is the initial enzyme activity (U), *Mf* is the enzyme activity of the filtrate (U) after immobilization, and *W* is the weight of wet gel beads (g). The best formulation was used for the naringinase catalytic experiments. On the other hand, the immobilization efficiency (I.E.) has been also calculated using the following equation:

2 $${\text{I}}.{\text{E}}.\% \, = \, \left( {Mi/Mo} \right)*100,$$

where *Mo* is the initial enzyme activity (U), and *Mi* is the enzyme activity of the immobilized enzyme per gram gel beads (U).

Moreover, the immobilization yield (I.Y.) has been calculated from the following equation:

3 $${\text{I}}.{\text{Y}}.\% = \left( {C/A - B} \right)*100,$$

where *A* is the activity of free enzyme added, and *B* is the activity of remaining enzyme, whereas *C* is the activity of immobilized enzyme. For determination of the optimum loading time, known concentration of enzyme was incubated with gel beads for different periods of time ranged from 2 to 24 h. The data were normalized to 100 % relative activity. The relative activity at each time is expressed as a percentage of the 100 % activity.

### Evaluation of naringinase catalytic activity

Various factors were studied to evaluate the catalytic activity of both the free and immobilized naringinase such as reaction temperature, effect of pH, and thermal stability of the enzymes, as follows:

### Operation temperature

The optimum temperature for the free and immobilized naringinase was examined by incubating the reaction mixture for both free and immobilized enzymes at different temperatures ranging from 30 to 80 °C for 1 h. The optimum temperature has been taken as 100 % activity and the relative activity at each temperature is expressed as a percentage of the 100 % activity.

### pH profile

The optimum pH for the free and immobilized naringinase was examined by incubation of free enzyme and loaded beads at optimum temperatures for 1 h into 1 mL of 1 mM of naringin dissolved at pHs 3–7.5 using different buffers. The data were normalized to 100 % activity. The highest enzyme activity is expressed as 100 %, and each pH is expressed relatively as a percentage of the 100 % activity.

### Enzyme thermal stability

To prove the stability of the immobilized enzyme at high temperatures, the enzymes were incubated in the enzyme’s buffer solution for a period of 3 h at 50, 60, and 70 °C, and then they were examined for enzyme activity as above. The data were normalized to 100 % activity. The highest enzyme activity is expressed as 100 %, and each temperature is expressed relatively as a percentage of the 100 % activity.

### Naringin hydrolysis, determination of *K*_*m*_ and *V*_max_, shelf and operational stability of immobilized naringinase

To evaluate the efficiency of the immobilized enzyme, three main experiments were carried out. The naringin hydrolysis using the optimum conditions for the free and immobilized enzyme, the shelf stability and reusability of the immobilized enzyme.

### Naringin hydrolysis

Equal units, 900 U, of free and immobilized enzymes were incubated at 70 °C for 1 h at pH 4 into the assay mixture. Samples were withdrawn at interval times from 15 to 120 min and analyzed for naringin hydrolysis.

### *K*_*m*_ and *V*_max_ of free and immobilized naringinase

The Lineweaver–Burk plot (double reciprocal) method was used to obtain the Michaelis–Menten kinetic models adequate for the description of the hydrolysis of naringin by the free and the immobilized enzyme. Apparent *K*_*m*_ and *V*_max_ of free and immobilized naringinase were determined by plotting 1/[*S*] against 1/[*V*], respectively.

4 $$\left[ S \right]/Vo = 1/V_{ \hbox{max} } *\left[ S \right] + K_{m} /V_{ \hbox{max} },$$

where [*S*] is the substrate concentration (naringin), *Vo* is the initial enzyme velocity, *V*_max_ is the maximum enzyme velocity, and *K*_*m*_ is the Michaelis constant and is defined only in experimental terms and equals the value of [*S*] at which *Vo* equals 1/2*V*_max_. The assay mixture comprised 900 U of free and immobilized enzyme, a substrate concentration of 29–230 mM at 70 °C, and pH 4 for 1 h.

### Free and immobilized enzymes’ shelf stability

The shelf stability was studied for the free and the immobilized enzyme over a period of 10 weeks at 4 °C. Ten grams of the immobilized enzyme containing 900 U and their equivalent of the free enzyme (900 U/mL) were stored in 0.1 M of acetate buffer (pH 4) at 4 °C. The samples were covered to avoid dehydration and loss of the buffer. A sample of the free enzyme (1 mL) or the immobilized enzyme (1 g gel beads) has been withdrawn every week and assayed for enzyme activity. The starting operational activity was considered as 100 % relative activity, and data were normalized to 100 % activity.

### Operational stability

The reusability of immobilized naringinase was studied using the novel grafted gel beads. The best conditions obtained from the optimum pH, temperature, and hydrolysis of naringin were used. One gram of the grafted gel beads was added to 2 ml of 0.2 % naringin solution in 0.1 M sodium acetate buffer, pH 4.0, The mixture was incubated for 1 h at 70 °C in a shaking water bath, and the substrate solution was assayed as above. The same gel disks were then washed with sodium acetate buffer and re-incubated with another substrate solution; this procedure was repeated for 20 times, and the initial activity was considered as 100 %. The relative activity was expressed as a percentage of the starting operational activity.

## Results and discussion

### Grafted alginate elucidation structure

**Fig. 1:**
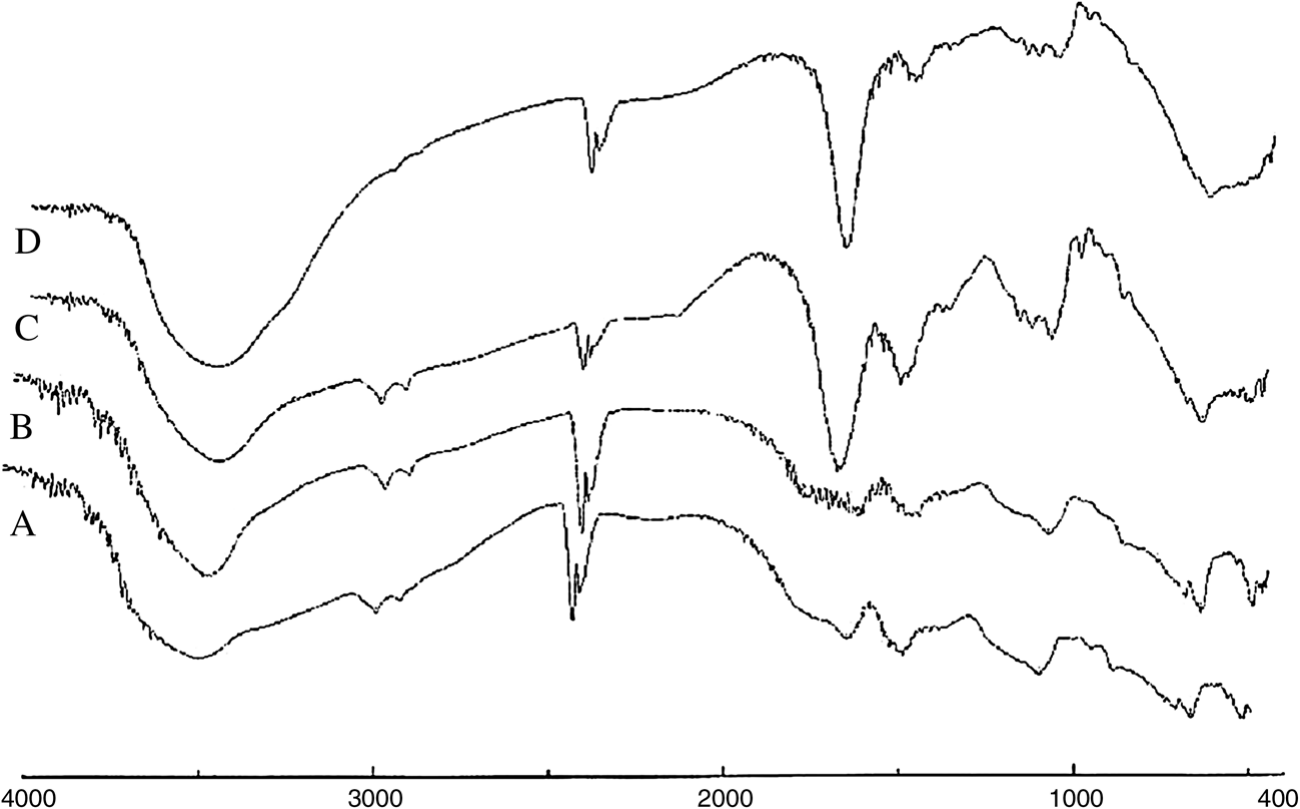
FTIR of *A* calcium alginate (Alg), *B* alginate hardened with calcium and polyethylenimine (Alg/PE + Ca^2+^), *C* alginate and polyethylenimine/Ca^2+^ beads followed by glutaraldehyde (Alg/PE + Ca^+2^/GA). *D* The interaction between the grafted alginate beads and naringinase

The FTIR spectroscopic analysis of Alg gel beads, grafted beads, and immobilized one were carried out from 400 to 4000 cm^−1^, as shown in Fig. 1. The FTIR bands of the Alg/Ca^2+^ (spectrum A) showed characteristic functional groups (–COO^−^ stretching) were present, with a broad asymmetrical band at 1610 cm^−1^ and a narrower symmetrical band at 1420 cm^−1^. The spectra for aminated beads showed a new broad peak at 3429 cm^−1^ which is corresponding to NH_2_ group that indicated the presence of amine group on the surface of beads (spectrum B), while glutaraldehyde activated beads showed new two peaks. The first of them is at 1717 cm^−1^ which refers to the (C=O) group of a free aldehyde end of glutaraldehyde, and the another peak is at 1660 cm^−1^ which is corresponding to Schiff’s base (C=N–) group which is the result of reaction of NH_2_ end groups with glutaraldehyde (spectrum C). Finally, the immobilized beads gave broader peak at 3457 cm^−1^, (spectrum D) indicating an increase in the concentration of NH_2_ groups which was found naturally in the enzyme. From all above, we can confirm that the process of amination, activation, and immobilization took place successfully. This result is in agreement with other published results (Tanaka et al. 1984).

**Fig. 2:**
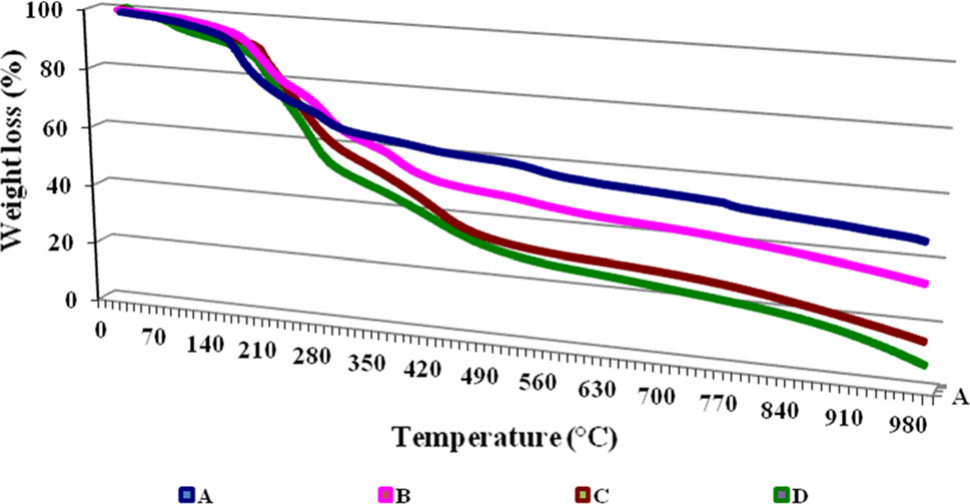
TGA thermographs of alginate gel beads (*A*), aminated beads (*B*), activated beads (*C*), and immobilized one (*D*)

The TGA thermo-gram of gel beads is shown in Fig. 2, and data are tabulated in Table 1. The treatment of Alg with PEI followed by GA showed a gradual and obvious improvement in their TGA. The TGA of Alg was at 200 °C compared to Alg/PEI which was at 230 °C and that of Alg/PEI/GA increased to 250 °C, and finally, after immobilization it shifted to 300 °C.

**Table 1:** TGA results of the gel beads formation steps and immobilization of naringinase

Type	TGA, °C
Alginate	200
Alginate + polyethyleneimine	230
Alg + PEI + glutaraldehyde	250
Alg + polyethyleneimine + glutaraldehyde + enzyme	300

The gels’ thermal improvement could be explained by the formation of polyelectrolyte interaction between the polyanions (–COO^−^) of alginate and the polycations (–NH_3_^+^) of the PE. Further hardening of the gel beads using GA could be attributed to the formation of a stronger cross linking of the gel beads due to formation of Schiff’s base between the free PEI’s amino groups and GA (Elnashar et al. 2009).

### Immobilization and optimization of the enzyme loading capacity (E.L.C.)

Data of the E.L.C. as well as the immobilization efficiency are tabulated in Table 2. Data showed that the E.L.C. increased gradually by 26-fold from 32 U/g gel to reach 853 U/g gel by increasing the enzyme concentration to zero dilution. This increase is due to gradual enzyme loading reaching its saturation at no enzyme dilution (Elnashar et al. 2009). According to Table 3, we achieved an enzyme loading of 899 U/g gel beads after only 12 h of incubation. The enzyme immobilization efficiency percent increased gradually by increasing the loading time reaching its maximum of 99 % with immobilization yield of 88 % of the total enzyme units after 12 h of contact time. Further soaking of the beads in contact with the enzymes has no positive effect and in some cases it reduced the enzyme activity. These results can be explained by assuming that the increase in contact time between the enzyme and the support created more bonds per enzyme molecule. Due to possible structure deformation during immobilization, the enzyme molecules can orientate themselves to remain with the active site blocked, thereby restraining the accessibility of the enzyme active site toward the substrate. Also, the longer incubation time could increase the probability for covalent modification close to the active site increasing enzyme inactivation (Costa et al. 2001).

**Table 2:** Optimization of the enzyme loading capacity using grafted alginate beads

Enzyme concentration percent (%)	Total enzyme units (soaking solution)	Immobilized enzyme U/g beads	(E.L.C.)	Immobilization yield % (I.Y. %)
100	900	868	835	96
75	675	610	558	90
50	450	398	318	88
25	250	210	180	84
10	80	60	32	75

**Table 3:** Optimization of the enzyme loading time using grafted alginate beads

Different loading times (h)	Total enzyme units (soaking solution)	Immobilized enzyme U/g beads	Immobilization efficiency % (I.E.%)	Immobilization yield % (I.Y.%)
2	900	100	11	61
4	900	233	25	29
6	900	400	44	11
8	900	600	66	22
10	900	876	97	86
12	900	899	99	88
14	900	830	92	81
16	900	876	97	86
18	900	890	98	87
20	900	888	98	87
22	900	876	97	86
24	900	889	98	87

### Effect of temperature and pH on naringinase activity

**Fig. 3:**
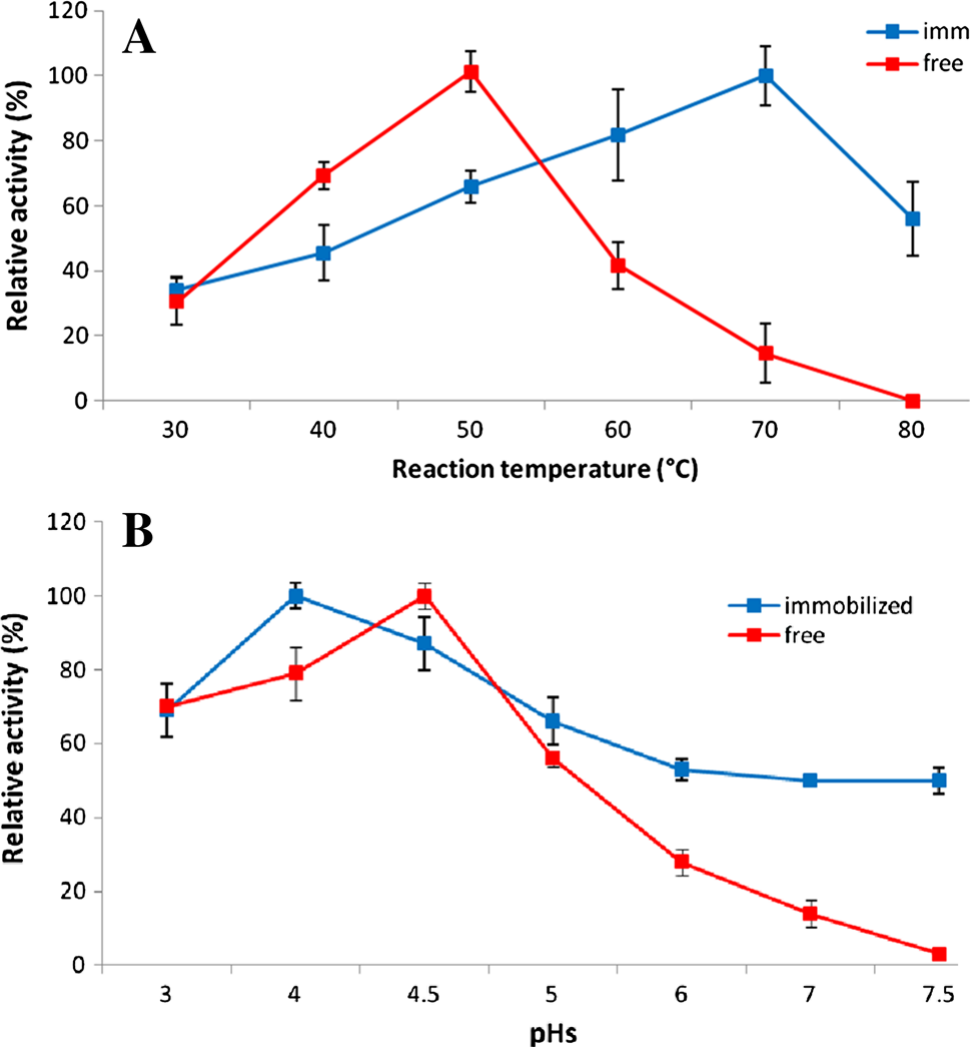
**a** Temperature profile of the free and immobilized naringinase enzyme. **b** pH profile of the free and immobilized naringinase enzyme

One of the main goals of this article was to improve the enzyme’s thermal stability to be suitable for industrial use as previously mentioned in the introduction. As shown in Fig. 3a, temperature of the free enzyme was at 50 °C and that of the immobilized enzyme was at 70 °C. This fact was supported by Chao et al. (1986) and Brennan (2002), by providing that only polyamines substantially improved hydrogel’s thermal stability. This shift of the enzyme’s optimum temperature after immobilization could be regarded to the formation of a molecular cage around the protein (enzyme), which protected the enzyme’s molecules from the bulk temperature. The optimum temperature of around 70 °C for hydrolysis of naringin by naringinase increased the potential for immobilized naringinase to be used on an industrial scale (Vila-Real et al. 2010). The optimum pH values for the free and the immobilized naringinase were very close as they were at pH 4.5 and pH 4, respectively. The immobilized naringinase displayed a higher retention of activity at pH 4 when compared with the free enzyme Fig. 3b. The shift to lower pH values is commonly found in immobilization of naringinase (Ellenrieder et al. 1998; Chang et al. 2011). However, the immobilized enzyme showed higher retention of activity compared to the free enzyme in most pH values. For example, at pH 7.5, the immobilized enzyme retained 60 % of its activity, whereas the free one completely lost its activity.

Due to the fact that most of the fruit juices that can be treated with naringinase normally show acidic pH values, this shift toward lower pH is an important improvement when considering the possible application of this immobilized system to the treatment of fruit juices.

### Thermal stability of the free and immobilized naringinase

**Fig. 4:**
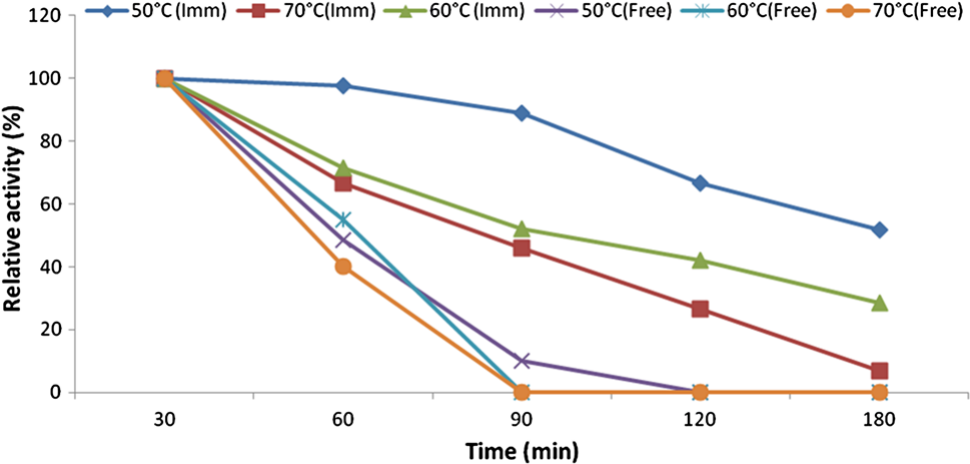
Temperature-stability profile of free and immobilized naringinase at 50, 60, and 70 °C

The thermal stability of the free and immobilized enzymes is shown in Fig. 4. The temperature stability of the free and immobilized enzymes at 50–70 °C revealed that the immobilized form is more stable for a longer time than the free enzyme. For example, at 50 °C, and for an incubation period of 120 min, the immobilized enzymes retained 66 % of its relative activity, whereas that of the free enzymes completely lost its activity. At 60 °C, the temperature at which the enzymes are preferably used in industries to avoid microbial contamination, the immobilized enzymes retained 52 % of their relative activity at 90 min, whereas the free one completely lost its activity. At 70 °C, the immobilized enzyme still retained 46 % of its relative activity after 90 min. In general, the decrease of enzymes’ activity might be due to the disturbance of globular structure of the protein by heat.

On the other hand, the immobilization process stabilized more of the 3D structure of the enzyme; in addition, the polymeric material for immobilization forms a cage surrounding the enzyme to protect it from the outside heat. In brief, the immobilized preparations were more stable than the soluble enzymes at higher temperatures and that is favorable in industries (Elnashar and Hassan 2014). Furthermore, the significant thermal stability of the immobilized naringinase could be resulted from the inside porous structure and microenvironment of the supports which prevented some intermolecular or protein–surface interactions (Le-Tien et al. 2004; Lei et al. 2011).

### Naringin hydrolysis using the free and immobilized naringinase

**Fig. 5:**
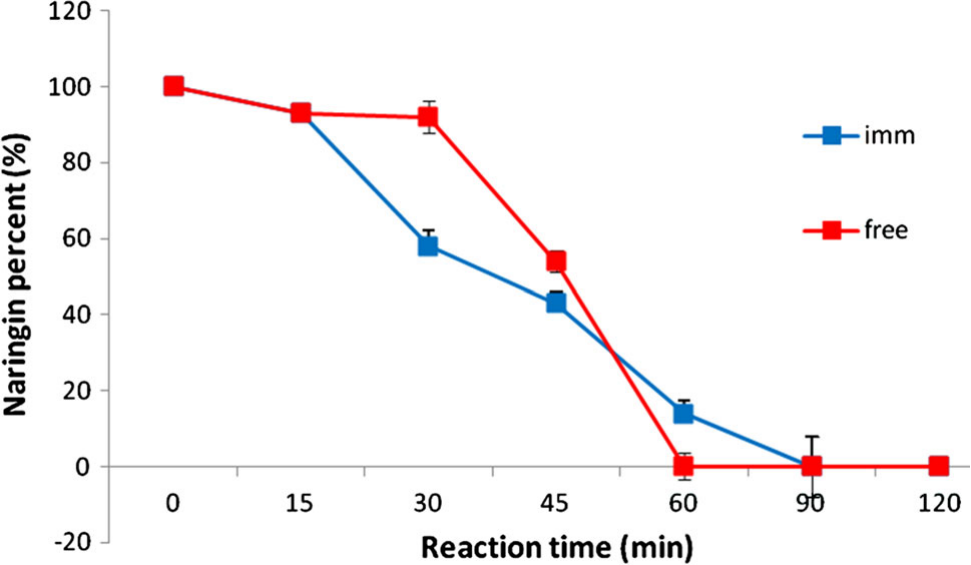
Hydrolysis of naringin using the free and immobilized naringinase

The results of the hydrolysis of naringin using the free and immobilized naringinase are shown in Fig. 5. At zero time, we had 100 % naringin. At 45 min, the free enzyme hydrolyzed 50 % of naringin compared to 40 % for the immobilized enzyme. However, 100 % hydrolysis of naringin was obtained for both the free and immobilized enzymes at 60 and 90 min, respectively. This close result between the free and immobilized enzymes could be regarded too little to no diffusion limitation has encountered the product substrate conversion using the immobilized enzyme (Elnashar et al. 2013). These results were in accordance to Yalim et al. (2004) who found that less than 5 % naringin was left after 86–97 min.

### Michaelis constant of free and immobilized naringinase

**Fig. 6:**
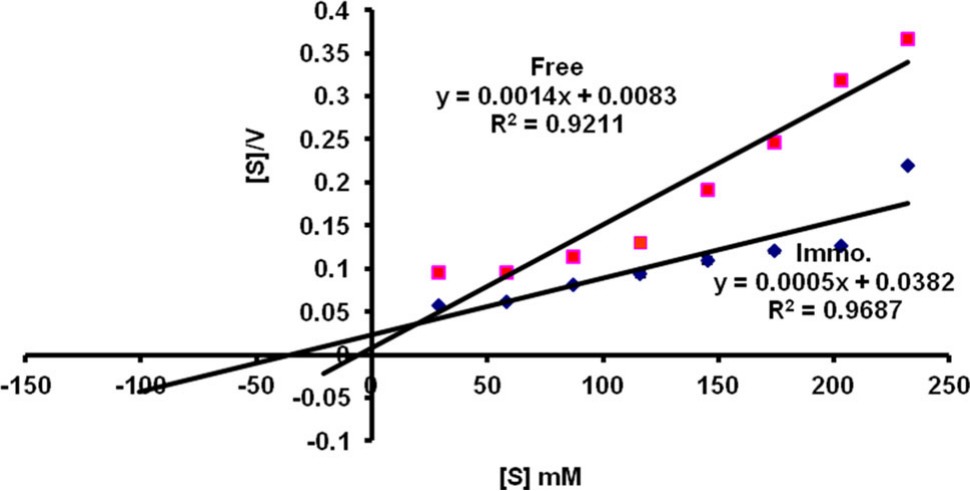
Michaelis constant of free and immobilized naringinase using Lineweaver–Burk plot method

The Michaelis constant of the free and immobilized naringinase was calculated using the double reciprocal plot method (Lineweaver–Burk plot) as shown in Fig. 6. The calculated *K*_*m*_ for the free enzyme was 5.9 mM, and similar result was obtained by Ellenrieder et al. (1998) and Soria et al. (2004). After immobilization, the apparent *K*_*m*_ increased to 41 mM indicating that a higher concentration of substrate is needed for the immobilized enzyme as the immobilized enzymes are not accessible as the soluble enzymes to the substrates. The maximum reaction velocity “*V*_max_” values for the immobilized enzyme were astounding: it increased from 8.3 mmol min^−1^ L^−1^ for free enzyme to 38.2 mmol min^−1^ L^−1^ for the immobilized one. This suggests that the native conformation of the enzyme is not altered at all after immobilization (Gopinath and Sugunan 2006). The increase of the enzyme’s *V*_max_ after immobilization could be interpreted from an energetic point of view as follows: for catalysis to be efficient, the loss of entropy, arising out of the initial binding of substrate to enzyme to form the enzyme–substrate complex, must be paid for by the binding energy released from them favoring interaction between enzyme and substrate (Chang et al. 2011). This implies that the loss of entropy, on forming the enzyme–substrate complex acts to increase its dissociation constant. Thus, weak binding of substrate to enzyme presents the enormous catalytic advantage of the intermolecular effect. In general, the behavior of increased *V*_max_ after immobilizing enzymes has been reported by many authors (Tanriseven and Dogan 2002; Roy et al. 2003).

### Operational and shelf stability of immobilized naringinase

**Fig. 7:**
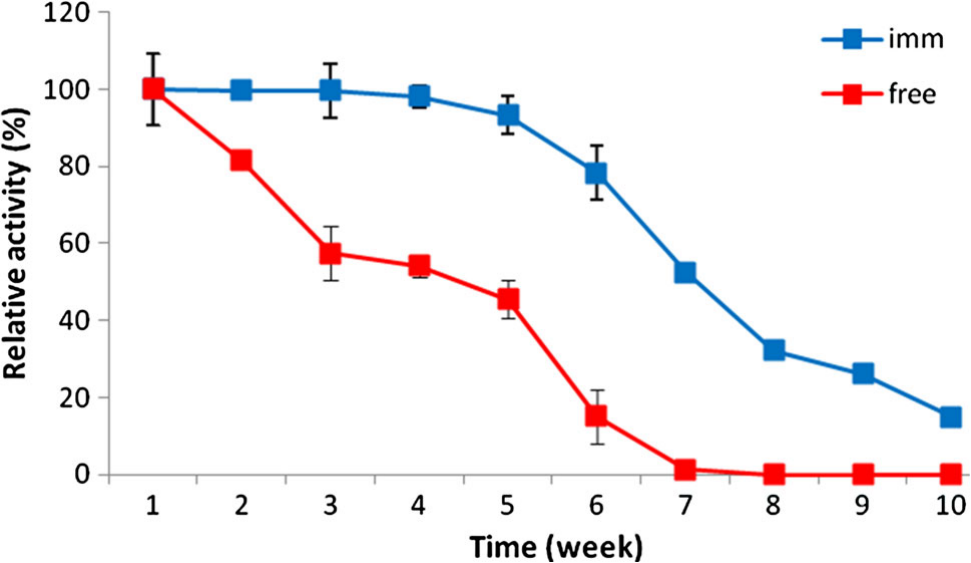
Shelf stability of free and immobilized naringinase at 4 °C

The shelf stability of the free and immobilized enzymes was studied at 4 °C for 10 weeks as shown in Fig. 7. The free enzyme lost about 20 % of its activity after 2 weeks, and after that, it declined to 43 % after 5 weeks and dramatically to 13 % after 7 weeks. On the other hand, the immobilized enzyme retained full activity for over 5 weeks, which is very useful for marketing the immobilized enzyme. Similar result was obtained by Ellenrieder et al. (1998) who immobilized naringinase by covalent binding to woodchips and the results showed that the immobilized enzyme retain 100 % of its activity for 30 days. The extended stability of the immobilized form could be attributed to protection of the 3D structure of the enzyme biomacromolecules onto the biopolymer beads.

**Fig. 8:**
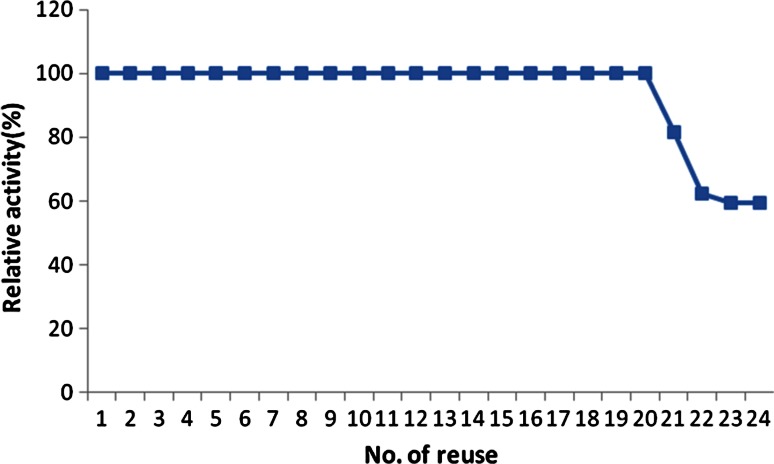
Operational stability of immobilized naringinase

The main advantage of immobilization of enzymes is the easy separation and reusability. The data shown in Fig. 8 indicated that the immobilized naringinase retained over 100 % of its activity after 20 reuses. The retention of the enzyme activity for almost 20 cycles with no loss of activity is a proof that the enzymes were immobilized to the gel beads via covalent bonds. Otherwise, if some enzymes were immobilized via physical bonds, they will not tolerate the reusability test and they would be lost. These results were far better than the one by Ribeiro and Rabaça (2011). The former succeeded in retaining 85 % of the initial activity of immobilized naringinase by cross-linking enzyme aggregates after three cycles; however, the enzyme activity dropped to 18 % by the 6th cycle. The slight decrease in the immobilized enzyme activity by the 20th use using the grafted alginate gel might be attributed to inactivation of enzyme due to continuous use.

## Conclusion

In this work, immobilization of naringinase onto grafted alginate beads revealed a significant improvement of the gel’s thermal stability from 200 to 300 °C. The enzyme loading capacity increased gradually to 28-fold of increase after which the carrier reached its saturation with enzymes. The results of optimization of pH values and temperatures were favored for the immobilized form. The shelf and operational stabilities showed outstanding results, retention of 100 % of the immobilized enzyme after 5 weeks and 20 reuses, compared to other authors who achieved only 16 % of the enzyme activity after the 6th use. These results are very useful on the marketing and industrial levels. Further studies could be also carried out to scale up the production of immobilized naringinase for the semi-pilot scale.
